# Low-flow CO_2_ removal integrated into a renal-replacement circuit can reduce acidosis and decrease vasopressor requirements

**DOI:** 10.1186/cc12833

**Published:** 2013-07-24

**Authors:** Christian Forster, Jens Schriewer, Stefan John, Kai-Uwe Eckardt, Carsten Willam

**Affiliations:** 1 Department of Nephrology and Hypertension, Friedrich-Alexander-University Erlangen-Nuremberg, Ulmenweg 18, Erlangen, 91054, Germany

**Keywords:** Lung-protective ventilation, Low-flow CO_2_ removal, ARDS, AKI, Renal-replacement therapy

## Abstract

**Introduction:**

Lung-protective ventilation in patients with ARDS and multiorgan failure, including renal failure, is often paralleled with a combined respiratory and metabolic acidosis. We assessed the effectiveness of a hollow-fiber gas exchanger integrated into a conventional renal-replacement circuit on CO_2_ removal, acidosis, and hemodynamics.

**Methods:**

In ten ventilated critically ill patients with ARDS and AKI undergoing renal- and respiratory-replacement therapy, effects of low-flow CO_2_ removal on respiratory acidosis compensation were tested by using a hollow-fiber gas exchanger added to the renal-replacement circuit. This was an observational study on safety, CO_2_-removal capacity, effects on pH, ventilator settings, and hemodynamics.

**Results:**

CO_2_ elimination in the low-flow circuit was safe and was well tolerated by all patients. After 4 hours of treatment, a mean reduction of 17.3 mm Hg (−28.1%) pCO_2_ was observed, in line with an increase in pH. In hemodynamically instable patients, low-flow CO_2_ elimination was paralleled by hemodynamic improvement, with an average reduction of vasopressors of 65% in five of six catecholamine-dependent patients during the first 24 hours.

**Conclusions:**

Because no further catheters are needed, besides those for renal replacement, the implementation of a hollow-fiber gas exchanger in a renal circuit could be an attractive therapeutic tool with only a little additional trauma for patients with mild to moderate ARDS undergoing invasive ventilation with concomitant respiratory acidosis, as long as no severe oxygenation defects indicate ECMO therapy.

## Introduction

In modern intensive care medicine, patients with multiorgan failure frequently need respiratory ventilation and renal-replacement therapy to bridge organ dysfunction. Ventilation itself, in particular with the use of high tidal volumes and high airway pressures, has been shown to be deleterious for patient outcomes [1,2], and thus protective ventilation strategies, including lower tidal volumes, have been implemented into clinical practice [1,3]. Although these ventilation specifications often lead to respiratory acidosis, the concept of permissive hypercapnia and concomitant acidosis is presently widely accepted, albeit still controversially discussed in the scientific community. Whereas evidence has been provided for immunologic, redox, and vasoactive protective effects, acidosis has also been associated with higher hemodynamic instability [4-7]. Extracorporeal membrane oxygenation (ECMO) [2,8-10] and pumpless CO_2_-removal systems (PECLA) [11,12] are increasingly used to support lung-protective ventilation strategies and to improve CO_2_ removal and respiratory acidosis. Importantly, in the recent Xtravent study by Bein *et al.*[13], pumpless CO_2_ removal enabled efficient low-tidal ventilation (about 3 ml/kg PBW) without severe acidosis, which was also associated with more ventilator-free days for patients having a severe oxygenation deficit (PaO_2_/FIO_2_ <150). However, in all of these therapies, a higher extracorporeal blood-flow circuit with additional risk and trauma by using special cannulas with big diameters to the patient is mandatory.

About 35% to 60% of the patients undergoing respiratory therapies in multiorgan failure also need renal-replacement therapies (RRTs) [14,15]. Because, in these patients with concomitant renal failure, extracorporeal blood circuits have necessarily already been established for renal-replacement therapy, we wondered whether addition of a hollow-fiber gas exchanger to the low-flow blood circuit could support lung-protective strategies by improving respiratory acidosis. Although renal-replacement circuits allow only a blood flow of 300 to 500 ml/min, partial elimination of CO_2_ appears to be feasible [16]. Arterial blood with a pCO_2_ of 40 mm Hg (5.3 kPa) contains approximately 500 ml CO_2_/L (pH 7.4). In sheep experiments, Young *et al.*[17] achieved 130 to 180 ml CO_2_ elimination (500 ml/L pCO_2_ in blood at 40 mm Hg (5.3 kPa) calculated) by using blood-flow rates of about 500 ml/min, combining a hollow-fiber gas exchanger and a hemofiltration device. Livigni *et al.*[18] also tested effects of low-flow CO_2_ elimination in sheep by using a veno-venous pump-driven bypass. They found an average CO_2_ reduction of hypercapnic ventilated sheep of 17% to 22%. Batchinsky *et al.*[19] were able to achieve normocapnia in hypercapnic ventilated pigs by using a veno-venous pump-driven system (400 to 600 ml/min blood flow), including a gas exchanger. Altogether, experimental evidence suggests, therefore, that a significant amount of basal CO_2_ production can be eliminated in low-flow veno-venous systems. First experiences in critically ill patients applying CO_2_ removal with low-flow veno-venous systems were gained by using a specialized device with a hollow-fiber gas exchanger adapted to low blood flows (about 350 ml/min; *“*DecapSmart”). First, case reports described successful application of this system in single patients [20-23]. Eventually, a clinical study using the DecapSmart system was able to demonstrate effects of low-flow CO_2_ removal in critically ill patients. Here in 10 patients being ventilated with a plateau pressure between 28 and 30 cm H_2_O and having respiratory acidosis, additional low-flow CO_2_ removal reduced pCO_2_ from 73.6 to 50.4 mm Hg and increased pH from 7.20 to 7.32 in 60 to 90 minutes [24]. However, the DecapSmart system still needs a specialized device and cannulation, which needs vascular access side to side with cannulas needed for renal-replacement therapy in severe AKI. We thus wondered whether implementation of a hollow-fiber gas exchanger into the renal circuit could combine low-flow CO_2_ removal and renal-replacement therapy by using one system and blood access and tested for ventilatory and hemodynamic effects in 10 severely ill patients with combined respiratory and renal failure.

## Materials and methods

### CRRT circuit

We used a CVVHD device (bm11/14; Edwards-Lifescience, Irvine, CA, USA) with a standard setup and adjustment for continuous venovenous hemodialysis. A high-flux polysulfone capillary hemofilter with a membrane surface area of 1.4 m^2^ (Polyflux 140 H; Gambro, Hechingen, Germany ) was used.

**Figure 1:**
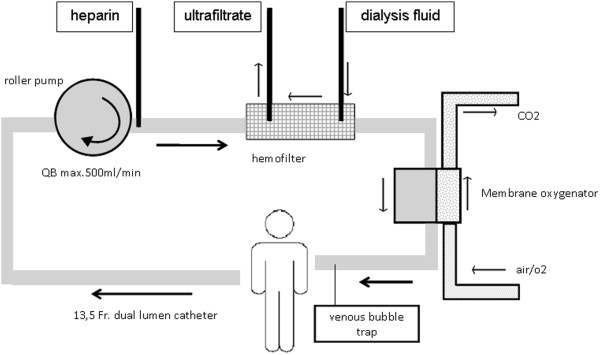
Scheme of the renal circuit with implementation of a hollow-filter gas exchanger.

For decarboxylation, a small standard hollow-fiber gas exchanger (D902 Liliput 2 ECMO; Sorin Group Milan, Italy) was applied. This gas exchanger has a surface area of 0.67 m^2^ and is intended for extracorporeal circuits with a maximum blood flow of 2,300 ml/min. According to the manufacturer’s description, the filter is coated with phosphorylcholine, which should form a phospholipid-like structure and reduce coagulation. The gas exchanger was integrated into the continuous hemodialysis system after the dialysis filter. For the connection, we used standard tubes with a Luer-lock system. The gas exchanger was attached to the hemodialysis machine with a conventional clamp (Figure 1). Venous and arterial pressures were monitored continuously. Gas flow through the gas exchanger was set to 4 L/min (blood flow, <300 ml/min) or 4 to 6 L/min (blood flow, >300 ml/min) with a FiO_2_ of 0.21. Only in cases in which oxygenation by the ventilator was critical, the FiO_2_ was varied (0.5 to 1.0 FiO_2_), in the hope of counteracting systemic hypoxia. For reasons of simplicity, we called this setting “lung-assisting renal replacement system” or “LARRS.”

The LARRS system was tested beforehand in “dummy” experiments, yielding no detectable changes in venous, arterial, and transmembrane pressures and confirming the full function of alarms, bubble catcher, and emergency shutter. In particular, flushing the system with air by artificial occlusion of all three ventilation tubes of the oxygenator led to prompt emergency standby of the CVVHD circuit, according to security standards in renal-replacement therapy devices. Also when the system was subsequently applied to patients, the pressure control of the hemodialysis did not show any differences in the presence of the gas exchanger in comparison to conventional renal-replacement therapy.

Blood flow was primarily adjusted to 400 ml/min, but could be adapted to individual needs and circumstances (300 to 500 ml/min). A 13.5-French double-lumen catheter was placed into the jugular vein. In two patients, we used a second 13.5-French double-lumen catheter in the femoral vein to allow higher blood flows. In this case, the two lumina of one catheter were linked by a y-adapter to form a single blood line. This allowed increasing the blood flow about 30% to 40% in these two patients. The dialysate contained Na, 140 m*M*; K, 4.0 *M*; Ca, 1.5 m*M*; Mg, 0.5 m*M*; Cl, 113 m*M*; HCO_3_, 35.0 mM; and D-Glucose, 9.0 g/L. Anticoagulation was performed with systemic heparin, and doses were prescribed targeting a PTT of 60 seconds or an activated clotting time (ACT), which was measured in bedside assays of 160 to 200 seconds. To prevent cooling of the patient, we used a tube heating system (Fresenius, Germany), which was set to 37°C.

### Patient inclusion

Patients were treated with the hollow-fiber gas exchanger in the renal circuit according to the individual decision of the treating physician, based on the patient’s characteristics and needs. Inclusion criteria were primarily the need for renal-replacement and mechanical-ventilation therapy with concomitant hypercapnic respiratory acidosis (pH <7.25). The treatment protocol was approved by the local Ethics Committee of the University of Erlangen-Nuremberg, Germany. In all cases, written informed consent was obtained from a legal guardian of the patient before application of the gas-exchange filter was started.

All patients had a central venous catheter in addition to the double-lumen catheters used for RRT, a urinary catheter (unless they were anuric), and an invasive blood-pressure measurement. Heart rate, blood pressure, SaO_2_, and temperature were monitored continuously; diuresis was measured hourly, and arterial blood gases were measured in variable intervals during the whole stay at the intensive care unit. Norepinephrine infusions were applied in parallel to fluid administration to maintain a mean arterial pressure of 65 mm Hg.

### Statistics

Because of the small number of patients, most results were presented for each case in absolute values or by calculating differences between baseline and 24 hours after commencing treatment. Means are expressed as mean ± SD of the mean.

## Results

### Patient characteristics

We treated 10 patients with the hollow-filter gas exchanger (LARRS) between November 2009 and January 2011. All patients were already undergoing CVVHD because of acute renal failure and oliguria when the CO_2_ hollow filter was applied. At inclusion, the arterial pH varied from 7.07 to 7.24; patient 7 was included to prevent progressive respiratory failure and exhaustion due to a still-compensated respiratory acidosis (HCO_3_, 31 m*M*; pCO_2_, 55 mm Hg; pH 7.37). The patients’ baseline characteristics are summarized in Table 1. Eight patients had community-acquired pneumonia; the other two patients had acute infectious exacerbation of COPD. Two patients (1 and 2) fulfilled ARDS criteria, but had contraindications against ECMO therapy. One patient (5) with severe ARDS was treated briefly for bridging until ECMO therapy was installed. Although this patient had preexisting high bicarbonate values (37 mm Hg) because of long-term adaptation to his COPD, this patient developed progressive respiratory acidosis (pH 7.34; pCO_2,_ 88 mm Hg) owing to his severe respiratory failure and increasing difficulties in ventilation support. The mean APACHE II score was 29.6 (APACHE II, 22 to 35), with a mean predicted mortality of 72% (40% to 82%).

**Table 1:** Baseline patients’ characteristics and outcomes of the 10 patients treated with the hollow-fiber gas-exchange device included in the renal-replacement circuit

**Number**	**Age**	**Gender**	**Height (cm)**	**Weight (kg)**	**BMI**	**pO**_**2**_**/FiO**_**2**_	**Apache II**	**Days on ventilation before LARRS**	**Diagnosis**	**Outcome**	**Comment**
1	51	M	180	90	35	95	31	11	H1N1- pneumonia	Died on ICU day 3	ECMO contraindication
2	47	M	178	75	23	91	34	1	H1N1- pneumonia	Died on ICU day 4	ECMO contraindication
3	57	F	160	55	17	141	28	13	Pneumonia	Weaning and recovery	
4	67	M	175	80	25	265	32	3	COPD	Weaning and recovery	
5	47	M	180	80	25	58	22	4	Pneumonia, COPD	Bridging to ECMO	Died on multiple organ failure 12 days later
6	74	M	170	90	31	93	35	1	Pneumonia	Weaning and recovery	
7	73	F	155	88	37	267	32	3	COPD	Weaning and recovery	
8	73	F	148	115	53	130	33	6	Pneumonia	Weaning	Died 25 days later – mesenteric ischemia
9	55	M	172	86	29	100	25	18	H1N1 – pneumonia	Weaning and recovery	
10	54	M	180	98	30	200	24	1	Pneumonia	Weaning and recovery	

### CO_2_ elimination in the CRRT circuit

Treatment modalities are summarized in Table 2. Application of the hollow-fiber gas-exchanger CVVHD system was well tolerated in all cases, and no change of function of the CVVHD device and the renal-replacement filter was detectable or necessary. Blood-flow rates from 250 ml to 500 ml could be achieved in all patients (mean, 378 ml/min at 24 hours). In particular, intraluminal pressures in the system were not altered. All patients tolerated the intervention well, and no complications occurred during the therapy. Mean heparin doses were 815 (*t* = 0 hours) and 1,116 IU/h (*t* = 24 hours), resulting in a mean PTT of 43 (*t* = 0 hours) and 72 (*t* = 24 hours) seconds. Mean activated clotting time was 177 (*t* = 0 hours) and 213 seconds (*t* = 24 hours) in bedside tests (see Additional file 1: Table S1). Two episodes of clotting were observed. In one case, the renal filter clotted after 23 hours of use and had to be exchanged. In a second case, the hollow-fiber gas exchanger clotted, which led to a rapid decrease of the arterial pH and increase of pCO_2_ in the extracorporeal system and activated pressure alarms of the dialysis machine. In this case, the hollow-fiber gas exchanger was exchanged immediately, which quickly restored CO_2_ removal and pH compensation.

**Table 2:** **Treatment modalities for low-flow CO**_**2 **_**removal integrated into the RRT circuit**

**No.**	**Double-lumen catheter**	**Blood flow (ml/min)**		**Dialysate flow (L/h)**		**Ultrafiltration (ml/h)**		**Volume (ml)**	**Gas flow (L/min)**	**Oxy FiO**_**2 **_**(vol. %)**	**Treatment time (hours)**
		***t*** **= 4 h**	***t*** **= 24 h**	***t*** **= 4 h**	***t*** **= 24 h**	***t*** **= 4 h**	***t*** **= 24 h**	***t*** **= 24 h**			
1	1	250	300	4.0	4.0	100	200	−645	6	1.00	30
2	1	330	400	2.0	2.0	200	0	−705	4	1.00	84
3	1	400	400	2.0	2.0	0	150	605	4	0.50	45
4	1	400	400	2.0	2.0	0	100	1,715	6	0.40	215
5	2	300^a^	n.a.	2.0 ^a^	n.a.	100 ^a^	n.a.	n.a.	6	0.21	2.5
6	1	500	450	3.0	3.0	0	100	1,340	6	0.21	191
7	1	320	250	2.0	2.0	100	150	−3,345	4	0.21	83
8	2	400	450	2.0	1.5	40	100	−2,055	6	0.21	71
9	1	400	500	2.0	2.0	0	0	180	6	0.21	92
10	1	250	250	2.0	2.0	50	0	2,945	4	0.21	134

Patient 5 was switched to ECMO after 2.5 hours of treatment and was therefore not applicable at *t* = 4 hours and *t* = 24 hours. ^a^t = 2.5 hours. n.a., not applicable.

Overall, in-device blood gas analysis directly after the hollow-fiber gas exchanger showed a mean reduction of extracorporeal pCO_2_ of 39 mm Hg (63 mm Hg before gas-exchanger to 24 mm Hg after gas-exchanger filter; see Additional file 2: Figure S1), resulting in a mean increase of pH of 0.31 (pH 7.28 before filter and 7.59 after filter, mean blood flow of 377 ml/min). The pO_2_ in the extracorporeal system increased from 41 mm Hg (before filter) to 122 mm Hg (after filter), but no change occurred in the patients’ systemic pO_2_. (Additional file 2: Figure S1).

### Blood-gas changes during the course of therapy

Implementation of the hollow-fiber gas exchanger reduced the average systemic arterial pCO_2_ by 17.3 mm Hg or 28.1% in about 4 hours (Table 3). The average pCO_2_ could then be kept constant during the next 24 hours. The pH concomitantly increased by 0.12 (0.04 to 0.19) in the first 4 hours, remaining approximately constant over the next 24 hours. No change was seen in arterial bicarbonate concentrations, presumably due to continuous dialysis against bicarbonate-containing dialysate. Despite an FiO_2_ of 1.0 in the gas flow to the gas-exchange device and a concomitant increase in the extracorporeal pO_2_ in the first two patients, no change occurred in their arterial pO_2_, which is consistent with the dominant role of pulmonary gas exchange for arterial pO_2_. The absence of a measurable effect on oxygenation can most likely be attributed to the low blood flow and thus to the low amount of oxygen provided for systemic circulation. We therefore subsequently reduced the FiO_2_ at the membrane oxygenator to 0.21 to avoid potential side effects or counterregulatory vascular effects in the pulmonary circulation arising from possibly increased oxygen tensions inside the pulmonary artery and pulmonary capillary bed. Despite the improvements in CO_2_ elimination, ventilator settings remained unchanged or were slightly deescalated. In particular, at *t* = 24 hours, mean P_max_ could be reduced from 32.50 to 28.67 mm Hg at *t* = 24 hours. Mean tidal volumes were slightly decreased from 8.41 to 7.34 ml PBW, eventually still not achieving 6 ml/kg PBW (Table 4).

**Table 3:** **Changes in systemic arterial pH, pCO**_**2**_**, bicarbonate (HCO**_**3**_**) from the beginning (*t*** **= 0 hours), 4 hours (*t*** **= 4 hours), and 24 hours (*t*** **= 24 hours) after starting low-flow CO**_**2 **_**removal**

**No.**	**Art. pH**			**Art. pCO**_**2**_			**Art. HCO**_**3**_		
	***t*** **= 0**	***t*** **= 4 hours**	**ΔpH**	**t = 0**	**t = 4 hours**	**ΔpCO**_**2**_	**t = 0**	**t = 4 hours**	**ΔHCO**_**3**_
1	7.17	7.25	0.08	58	47	−11	20	20	0
2	7.24	7.28	0.04	56	51	−5	22	24	2
3	7.10	7.29	0.19	77	49	−28	23	23	0
4	7.18	7.34	0.16	69	52	−17	25	27	2
5	7.24	7.21^a^	−0.03	88	90^a^	2	37	34^a^	−3
6	7.07	7.18	0.11	77	57	−20	20	19	−1
7	7.37	7.44	0.07	55	44	−11	31	30	−1
8	7.16	7.24	0.09	66	55	−11	23	23	0
9	7.18	7.31	0.13	79	55	−24	29	27	−2
10	7.18	7.37	0.19	65	36	−29	23	21	−2
**Mean**	**7.18**	**7.30**	**0.12**	**69.00**	**49.56**	**−17.3**	**25.3**	**23.78**	**−0.22**

Deltas indicate differences between *t* = 0 and *t* = 4 hours. Patient 5 was switched to ECMO after 2.5 hours of treatment and was therefore excluded from calculation of means and deltas at *t* = 4 hours. ^a^*t* = 2.5 hours.

**Table 4:** **Ventilator settings from the beginning (*t*** **= 0 hours), 4 hours (*t*** **= 4 hours) and 24 hours (*t*** **= 24 hours) after starting low-flow CO**_**2 **_**removal**

**Number**	**FiO**_**2 **_**(%)**			**PEEP (mm Hg)**			**Tidal volume (ml/pbw)**			**P**_**mean **_**(mm Hg)**			**P**_**max **_**(mm Hg)**			**Breathing rate**		
	***t*** **= 0 h**	***t*** **= 4 h**	***t*** **= 24 h**	***t*** **= 0 h**	***t*** **= 4 h**	***t*** **= 24 h**	***t*** **= 0 h**	***t*** **= 4 h**	***t*** **= 24 h**	***t*** **= 0 h**	***t*** **= 4 h**	***t*** **= 24 h**	***t*** **= 0 h**	***t*** **= 4 h**	***t*** **= 24 h**	***t*** **= 0 h**	***t*** **= 4 h**	***t*** **= 24 h**
1	0.85	0.95	1.00	14	14	14	8.6	8.0	8.6	22	22	23	32	32	32	25	23	20
2	1.00	0.95	1.00	14	16	20	7.5	5.8	5.1	22	22	23	32	32	30	22	22	30
3	0.55	0.55	0.50	10	10	10	8.3	8.2	7.6	19	18	19	35	32	34	30	28	28
4	0.40	0.40	0.35	8	8	8	8.2	8.2	7.2	17	17	15	32	31	27	20	20	20
5	1.00	1.00^a^	n.a.	13	13 ^a^	n.a.	8.6	6.5 ^a^	n.a.	23	23 ^a^	n.a.	40	40 ^a^	n.a.	20	24 ^a^	n.a.
6	1.00	0.95	0.60	12	12	12	9.0	8.8	8.0	20	19	19	34	33	31	20	20	20
7	0.37	0.37	0.37	12	12	10	9.6	9.5	8.1	16	14	13	27	23	21	12	12	14
8	0.50	0.50	0.50	15	15	15	9.8	8.4	6.7	21	21	21	33	33	30	20	20	26
9	0.80	0.70	0.60	12	12	14	7.9	9.6	7.7	19	19	18	28	28	25	28	28	24
10	0.40	0.40	0.50	12	12	12	6.6	8.6	7.1	19	19	18	32	32	28	26	26	26
**Mean**	**0.69**	**0.64**	**0.60**	**12.20**	**12.33**	**12.78**	**8.41**	**8.34**	**7.34**	**19.80**	**19.00**	**18.78**	**32.50**	**30.67**	**28.67**	**22.30**	**22.11**	**23.11**

n.a., not applicable. Patient 5 was switched to ECMO after 2.5 hours of treatment and was therefore excluded from calculation of means at *t* = 4 hours and *t* = 24 hours. ^a^t = 2.5 hours.

### Hemodynamic stability

**Figure 2:**
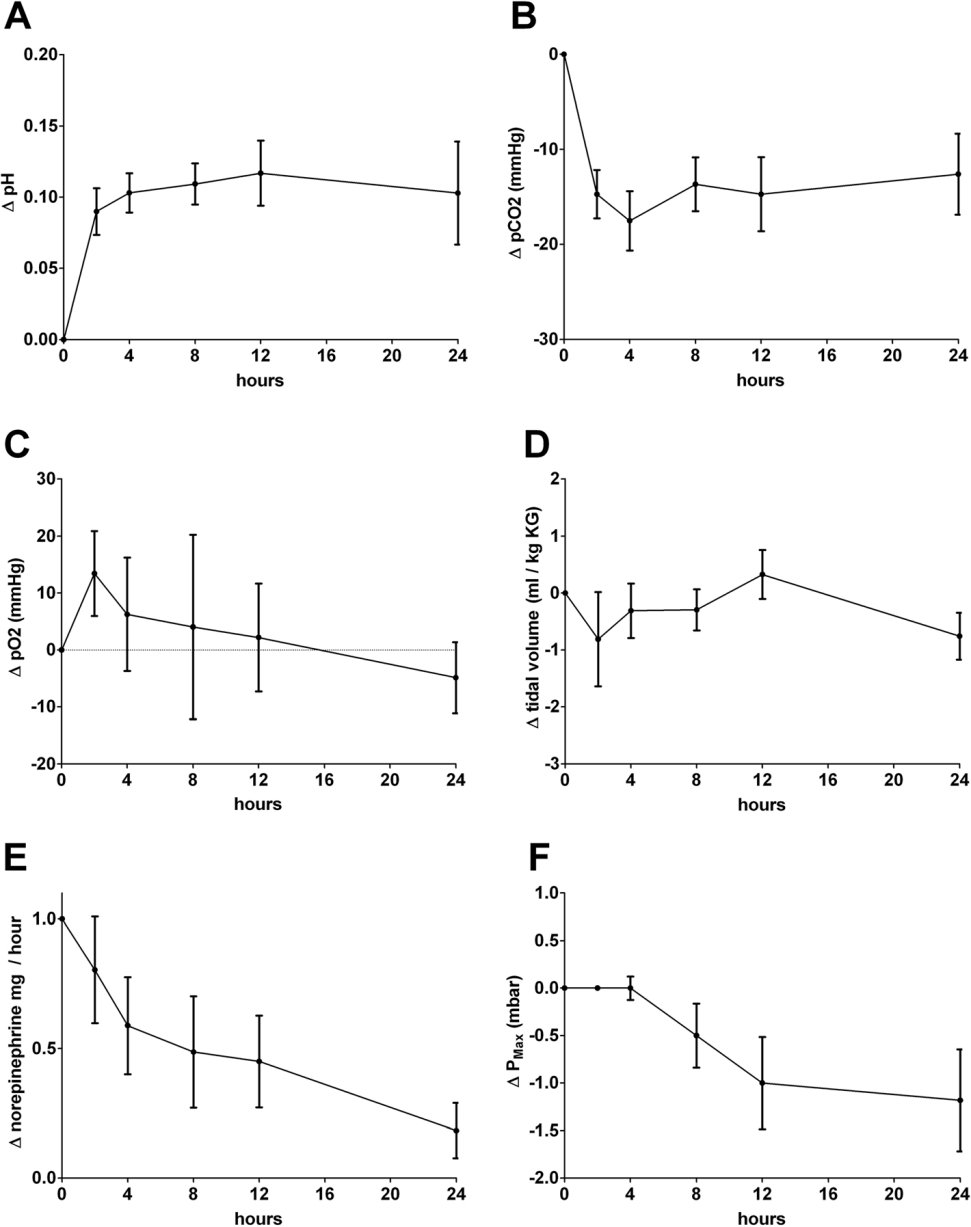
**Clinical effects of low-flow CO**_**2 **_**removal 4 and 24 hours after commencement of treatment.** Graphs illustrate mean changes of values for pH **(A)**, pCO_2_**(B)**, pO_2_**(C)**, tidal volume on respirator **(D)**, norepinephrine dose at mg/h **(E),** and average changes in P_max_ on the ventilator **(F)**.

**Figure 3:**
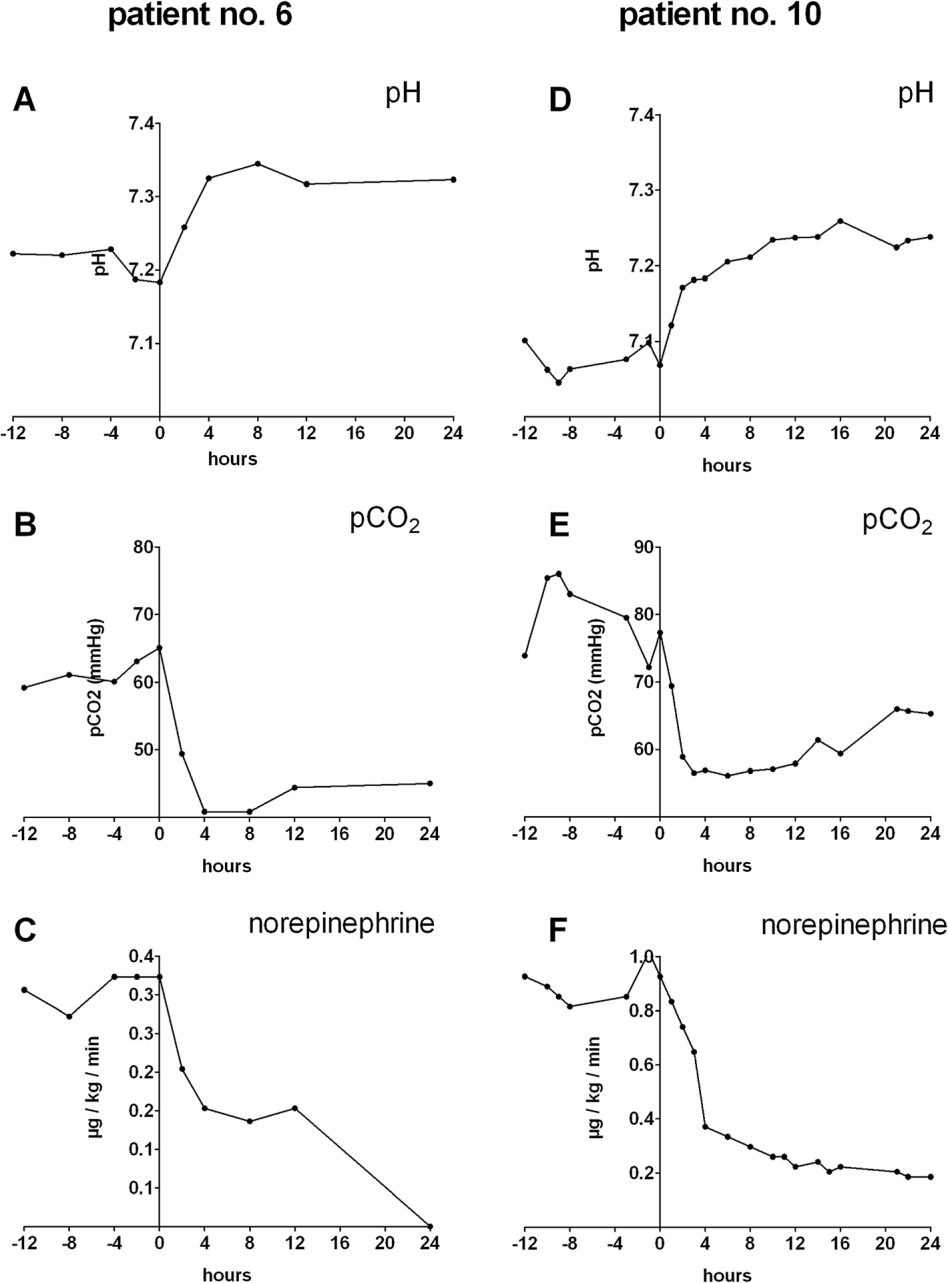
**Exemplary time courses for clinical effects of low-flow CO**_**2 **_**removal in two patients.** Patient 10 had acute pneumonia **(A**, **B**, **C)**; patient 6 had cardiomyopathy and acute pneumonia **(D**, **E**, **F)** for pH, pCO_2_, and norepinephrine dose, respectively.

In parallel to pH correction, a marked stabilization of hemodynamics was observed (Figure 2). When the hollow-fiber gas-exchanger CVVHD system was started, nine of 10 patients received norepinephrine therapy, five of these in doses between 0.6 and 5.0 mg/h (0.13 to 0.93 μg/kg/min). In four of these five patients with higher doses, a marked hemodynamic stabilization with decreased norepinephrine doses (average 65% reduction) could be achieved during the therapy (Table 5). In the remaining hemodynamically unstable patient (number 1), acidosis could not be corrected, and no hemodynamic stabilization occurred. In terms of volume control, patients were either ultrafiltrated because of volume overload in lung failure in the first 24 hours (four patients, -645 to −3,345 ml/24 hours) or received volume replacement as part of hypertensive shock therapy (six patients, 180 to 2,945 ml/24 hours). No obvious difference was seen in the response to CO_2_ elimination between patients in whom either negative or positive fluid balance was achieved. Exemplary time courses of pH, pCO_2_, and norepinephrine dose in two patients who were successfully treated with the low-flow RRT system are shown in Figure 3.

**Table 5:** **Hemodynamic parameters from the beginning (*t*** **= 0 h), 4 hours (*t*** **= 4 h), and 24 hours (*t*** **= 24 h) after starting low-flow CO**_**2 **_**removal**

**Number**	**MAP (mm Hg)**			**Heart rate**			**Norepinephrine (mg/h)**			**Norepinephrine (μg/kg/min)**			**Lactate (mg/dl)**		
	***t*** **= 0 h**	***t*** **= 4 h**	***t*** **= 24 h**	***t*** **= 0 h**	***t*** **= 4 h**	***t*** **= 24 h**	***t*** **= 0 h**	***t*** **= 4 h**	***t*** **= 24 h**	***t*** **= 0 h**	***t*** **= 4 h**	***t*** **= 24 h**	***t*** **= 0 h**	**t = 4 h**	***t*** **= 24 h**
1	83	73	66	120	110	100	2.6	3.2	5.2	0.48	0.59	0.96	49	59	124
2	57	73	80	120	110	125	0.2	0.2	0.4	0.04	0.04	0.08	12	12	4.2
3	80	87	73	80	85	90	0.2	0.0	0.0	0.06	0.00	0.00	7	9	6
4	80	80	87	110	105	100	0.6	0.5	0.3	0.13	0.10	0.06	14	16	18
5	73	76^a^	n.a.	110	100 ^a^	n.a.	0.2	0.3 ^a^	n.a.	0.04	0.06 ^a^	n.a.	11	9 ^a^	n.a.
6	70	77	93	110	90	90	5.0	2.0	1.0	0.93	0.37	0.18	43	47	12
7	70	73	90	70	65	95	0.1	0.0	0.0	0.02	0.00	0.00	6	7	9
8	66	63	73	110	90	90	1.2	1.2	0.9	0.17	0.17	0.13	12	13	14
9	80	82	73	105	110	110	0.0	0.0	0.0	0.00	0.00	0.00	7	7	8
10	73	87	93	100	85	80	1.9	0.9	0.0	0.32	0.15	0.00	10	12	9
**Mean**	**73.20**	**77.22**	**80.89**	**103.50**	**94.44**	**97.78**	**n.a.**	**n.a.**	**n.a.**	**0.22**	**0.16**	**0.16**	**17.10**	**20.22**	**22.69**

n.a., not applicable. Patient 5 was switched to ECMO after 2.5 hours of treatment and was therefore excluded from calculation of means at *t* = 4 hours and *t* = 24 hours.

^a^*t* = 2.5 hours.

### Patients’ outcome

No serious adverse events could be attributed to the hollow-fiber gas exchanger or the RRT circuit during treatment of the patients. Seven of 10 patients were successfully weaned from the low-flow CO_2_ removal system, improved their pulmonary function, and recovered from critical illness (Table 1).

In the two patients (patients 1 and 2), who had contraindications for ECMO therapy, low-flow CO_2_ removal was applied into the preexisting CVVHD devices as an additional limited therapy option in very severe multiple organ failure: both patients had H1N1 infection with pulmonary ARDS and very low oxygenation indices (95/91). Patient 1 had also a severe liver failure secondary to preexisting alcoholic liver cirrhosis Child C. He died at day 3 after study inclusion. Patient 2 had Hodgkin disease and was undergoing chemotherapy. He had acquired H1N1 and concomitant *Aspergillus fumigatus* pneumonia. We eventually limited intensive care support because of the overall poor prognosis and in line with the patient’s provision. He died at day 4 after commencing intensive care therapy. Patient 5, with ARDS due to pneumonia, was bridged to ECMO therapy for only 2.5 hours, before ECMO cannulas were applied and an ECMO device was available. He died 12 days later of multiorgan failure despite continued ECMO therapy.

## Discussion

For the first time we here report the use of a low-flow hollow-fiber gas exchanger implemented in a CRRT circuit in a small series of critically ill patients. The simple device proved to be efficient in terms of CO_2_ elimination, was well tolerated, and did not lead to adverse events. Concomitant renal-replacement therapy was in no way compromised, and alarm functions of the CRRT system ensured safety control for the gas-exchange device.

The concept of permissive hypercapnia has been developed to reduce baro- and volutrauma during ventilation. Increased CO_2_ levels have been shown to be associated with some potentially beneficial (for example, antiinflammatory effects), but the resulting acidosis also induces hemodynamic instability and potential cellular adverse effects [4-7]. In our case series in eight of 10 patients, application of the gas-exchange filter led to a rapid, partial, or complete correction of the pH and a significant reduction of the pCO_2_ within 4 hours. Ranges of CO_2_ reduction and pH correction were overall comparable to results obtained in the previous study of Terragni *et al*. [24] using a standalone low-flow CO_2_-removal system. In five of six hemodynamically unstable patients requiring higher doses of norepinephrine (>0.5 mg/h/>0.1 μg/kg/min), pH correction was in line with a marked reduction of vasopressor needs and an improved hemodynamic stability. Norepinephrine doses could be reduced to approximately one half after 6 to 8 hours on average; in three patients, vasopressors could even be stopped. Hemodynamic stability correlated with pH correction by enhanced CO_2_ elimination.

In the CRRT circuit used, blood flow was limited to 500 ml/min at maximum, provided that catheter flow was optimal. We here used a high ratio of blood to gas flow to achieve maximum CO_2_ elimination, although lower blood/gas-flow ratios would be potentially effective as well. Achieved CO_2_ removal allowed correction of acidosis, but we did not find a significant contribution to the systemic oxygenation of the patients even when higher FiO_2_ together with high gas/blood-flow ratios were applied. Thus, additional systemic oxygenation cannot be achieved with this low-flow device and is reserved for extracorporeal lung replacement therapies with higher flow rates, as in ECMO or PECLA therapy.

As it was a pilot, nonrandomized proof-of-concept study, our study has several inherent limitations. First, implementation of the device was one of multiple interventions in the severely ill patients under investigation. Treatment included volume administration, antibiotic treatment, and further co-medication to treat the underlying disease, which was sepsis in eight of 10 cases. Treatment of acidosis was not confined to CO_2_ elimination by the respirator and the hollow-fiber gas exchanger, but also included bicarbonate filtrate substitution and proton dialysis during the course of renal-replacement therapy. However, the time course of blood pH and pCO_2_ in individual patients with immediate responses after the implementation of the hollow-fiber gas exchanger clearly argue for a causal impact of the device. Moreover, metabolic acidosis was already balanced by bicarbonate dialysis, when low-flow CO_2_ removal was started, and bicarbonate levels remained more or less stable during renal-replacement therapy and therefore most likely did not contribute to the observed effects. Second, we did not apply a standardized ventilation protocol, which would have allowed testing for effects of the intervention on ventilatory requirements or achieving low tidal volumes of 6 ml/kg PBW or less. However, in our clinical setting, the hollow-fiber gas exchanger in a low-flow circuit CO_2_ at least compensated for respiratory acidosis and avoided further increases in ventilation settings or even led to slight reductions of peak pressure despite ongoing respiratory failure. Statistically, the overall survival in this small series was much higher than predicted by the calculated APACHE II scores of the patients, but a sample size of 10 patients certainly does not allow drawing any conclusions in respect to outcomes.

Despite this principal limitation of the hollow-fiber gas exchanger inserted into an RRT circuit, the system may have significant advantages in settings where the control of hypercapnia is desirable, including less-severe ARDS, when ECMO therapy is either still not indicated or simply not available. The advantages of applying a hollow-fiber gas exchanger in a CRRT circuit include its simplicity and its potential applicability in nonspecialized centers, which are experienced in renal-replacement therapy, as well as the fact that no additional catheter placements are needed. In contrast to many ECMO devices, available RRT machines are more secure and are equipped with distinct alarm systems, which allow emergency shut-off due to air or clots in the blood circuits. Regional anticoagulation with citrate was not performed in conjunction with the described setting, because of the necessity for increased blood flows, but regional anticoagulation protocols could possibly be developed, because a hemofilter allowing clearance of calcium-citrate complexes is included in the system, which is not the case in systems with isolated hollow-fiber gas exchangers.

## Conclusions

Implementation of a hollow-fiber gas exchanger in a low-flow CRRT circuit was feasible and safe and let to a significant CO_2_ removal and rapid correction of arterial pH in critically ill patients with acute renal and respiratory failure, with a positive impact on hemodynamic stability. Integration of a hollow-fiber gas exchanger could thus be potentially an additive tool in the armamentarium of treatment modalities in patients with multiorgan failure. To this end, additional, larger, and controlled studies are certainly needed to assess the impact of low-flow CO_2_ removal on ventilator settings and patient prognosis.

## Key messages

•Implementation of a hollow-fiber gas exchanger in a low-flow CRRT circuit was feasible and safe

•Low-flow CO_2_ removal in a CRRT circuit significantly removed CO_2_ and allowed rapid correction of arterial pH in critically ill patients with acute renal and respiratory failure

•Correction of arterial pH contributed to hemodynamic stabilization of the patient

•Low-flow CO_2_ did not significantly contribute to systemic oxygenation

•Low-flow CO_2_ removal complements but did not substitute ECMO or PECLA therapy, when ECMO/PECLA was indicated

## Abbreviations

ACT: Activated clotting time; AKI: Acute kidney injury; ALI: Acute lung injury; ARDS: Acute respiratory distress syndrome; CRRT: Continuous renal-replacement therapy; CVVHD: Continuous veno-venous hemodialysis; ECMO: Extracorporeal membrane oxygenation; LARRS: Lung-assisting renal-replacement system; PBW: Predicted body weight; PECLA: Pumpless extracorporeal lung assist; RRT: Renal-replacement therapy.

## Competing interests

Sorin (Milan, Italy) provided the hollow-fiber gas exchangers without cost. The authors declare that they have no competing interests.

## Authors’ contributions

CF participated in the design of the study, carried out the data acquisition and analysis, and participated in drafting the manuscript. JS primarily established the LARRS system and controlled the technical application. SJ and KUE participated in the design of the study and participated in drafting the manuscript. CW conceived of and designed the study, carried out the coordination, and drafted the manuscript. All authors read and approved the final manuscript.

## Supplementary Material

Additional file 1: Table S1 Anticoagulation therapy and clotting values after starting low-flow CO_2_ removal. n.a., not applicable. Click here for file

Additional file 2: Figure S1 In device pre- and postfilter pH, pCO_2_ and pO_2_ blood-gas measurements values. Click here for file
