# Supplementary material for: Low-flow CO2 removal integrated into a renal-replacement circuit can reduce acidosis and decrease vasopressor requirements
Source: Crit Care. 2013 Jul 24;17(4):R154. doi: 10.1186/cc12833 (PMC4056563; doi:10.1186/cc12833)
Supplement: Additional file 1: Table S1 — Anticoagulation therapy and clotting values after starting low-flow CO2 removal. n.a., not applicable. [file cc12833-S1.doc]

**Table S1:** Anticoagulation therapy and clotting values after starting low-flow CO_2_ removal. n.a.: not applicable

| **no.** | **heparin (IU)** | | | **activated clotting time (sec)** | | **partial thromboplastin time (sec)** | | **prothrombin time (%)** | | **thrombocytes (x 10^3^ /µl)** | |
| --- | --- | --- | --- | --- | --- | --- | --- | --- | --- | --- | --- |
|  | t=0 h | t=4 h | t=24 h | min | max | t=0 h | t=24 h | t=0 h | t=24 h | t=0 h | t=24 h |
| 1 | 800 | 720 | 720 | 200 | 242 | 59 | 98 | 24 | 17 | 46 | 30 |
| 2 | 800 | 800 | 720 | 230 | 263 | 42 | 43 | 75 | 75 | 12 | 38 |
| 3 | 500 | 700 | 1000 | 164 | 190 | 37 | 68 | 80 | 70 | 334 | 246 |
| 4 | 1000 | 1300 | 1100 | 152 | 239 | 41 | 121 | 57 | 48 | 328 | 254 |
| 5 | 500 | n.a. | n.a | 190 | 190 | 38 | n.a. | >100 | n.a. | 33 | n.a. |
| 6 | 1000 | 1000 | 1500 | 167 | 217 | 45 | 51 | >100 | >100 | 132 | 105 |
| 7 | 400 | 400 | 600 | 159 | 200 | 35 | 38 | >100 | >100 | 60 | 29 |
| 8 | 800 | 1000 | 1300 | 156 | 202 | 39 | 86 | >100 | >100 | 132 | 133 |
| 9 | 1250 | 1500 | 1800 | 159 | 194 | 43 | 54 | >100 | >100 | 81 | 72 |
| 10 | 1100 | 1300 | 1300 | 190 | 192 | 47 | 87 | 98 | >100 | 47 | 53 |
| **mean** | **815** | **968** | **1115** | **176** | **212** | **42.6** | **71.7** | **83.4** | **78.8** | **120.5** | **106.6** |
