# Supplementary figures and images for: Low-flow CO2 removal integrated into a renal-replacement circuit can reduce acidosis and decrease vasopressor requirements
Source: Crit Care. 2013 Jul 24;17(4):R154. doi: 10.1186/cc12833 (PMC4056563; doi:10.1186/cc12833)

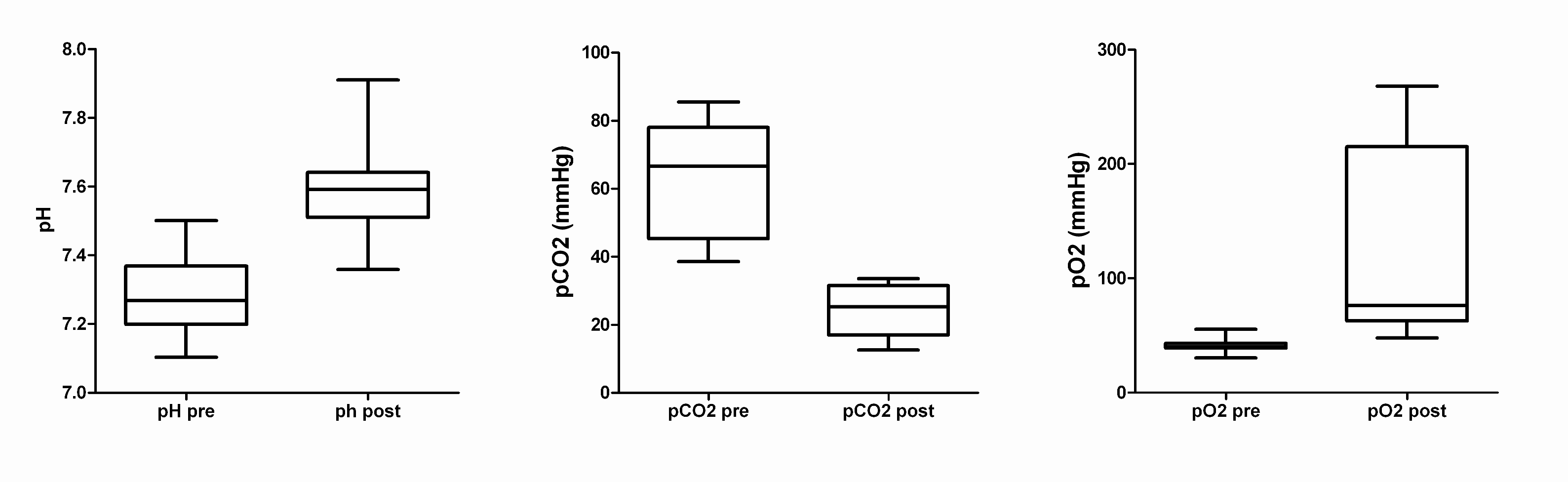

Supplement: Additional file 2: Figure S1 — In device pre- and postfilter pH, pCO2 and pO2 blood-gas measurements values. [file cc12833-S2.tiff]
